# Social Environment and Individual Differences in Feeding Behavior Are Associated with Risk of Endometritis in Dairy Cows

**DOI:** 10.3390/ani9100828

**Published:** 2019-10-19

**Authors:** Alexander Thompson, Kathryn L. Proudfoot, Becca Franks, Marina A.G. von Keyserlingk

**Affiliations:** 1 Animal Welfare Program, Faculty of Land and Food Systems, The University of British Columbia, Vancouver, BC V6T 1Z4, Canada; alexithompson42@gmail.com; 2 Veterinary Preventive Medicine, College of Veterinary Medicine, The Ohio State University, Columbus, OH 43210, USA; kproudy@gmail.com; 3 Department of Environmental Studies, New York University, New York, NY 10003, USA; beccafranks@gmail.com

**Keywords:** individual variation, predictability, social stress, animal welfare

## Abstract

**Simple Summary:**

The aim of this study was to determine how individual differences in behavior affect disease risk in Holstein dairy cows housed in two different social environments: (1) a predictable and non-competitive social environment and (2) an unpredictable and competitive social environment. Individual differences in feed intake and feeding behavior before calving were associated with cytological endometritis post-calving; however, the direction and magnitude of these effects were dependent on the social environment. These results provide the first evidence that individual differences in feeding behavior affect cytological endometritis risk differently depending on the social environment.

**Abstract:**

Our aim was to determine whether individual differences in feeding and social behavior in different social environments affect health outcomes in dairy cows. We used eight groups of four animals per treatment assigned to either a ‘predictable’ or an ‘unpredictable’ and competitive social environment. Predictable cows were given free access to six feed bins with no change in feed delivery times; whereas, the unpredictable cows were required to share one feed bin with one resident cow and morning feed was delayed 0, 1, 2, or 3 h every other day. On alternate days, the unpredictable cows were also re-assigned to a new bin and a new resident partner. Low daily dry matter intake (DMI) was a risk factor for cytological endometritis in predictable cows (odds ratio (OR) (95% confidence interval): 0.17 (0.02, 0.53)), but low daily DMI was protective for unpredictable cows (OR: 1.93 (1.09, 4.14)). Although low rate of DMI (kg/min) was a risk factor for cytological endometritis for predictable cows (OR: 4.2 × 10^−101^ (8.6 × 10^−206^, 4.8 × 10^−30^)) it was unrelated to disease for unpredictable cows. There were no associations between feed bin visits or percentage of non-nutritive visits with the likelihood of cytological endometritis. This is the first evidence that individual differences in feeding behavior influence cytological endometritis risk in dairy cows, but the direction and magnitude of these effects is dependent on the social environment.

## 1. Introduction

In the weeks before and after giving birth, intensively managed dairy cows commonly face a number of social stressors related to their management and environment. These stressors in combination with physiological and metabolic changes that occur after calving put dairy cows at high risk for developing disease during this time. LeBlanc et al. [1] estimated that 75% of disease occurs within one month of calving, with approximately 30%–50% of dairy cows experiencing some form of metabolic or infectious disease during this time [2]. Although there is substantial research on the early detection and treatment of the diseases that occur after calving, less research has attempted to identify characteristics of the ‘at-risk’ animal.

Work with pigs has shown that individuals that cope poorly with stressful events are at higher risk of disease compared to those that avoid or are able to cope with stressors [3]. In the human and laboratory animal literature, unpredictable and uncontrollable social factors have been found to be particularly damaging to health [4,5,6,7]. Similarly, in the farm animal literature, there are a growing number of studies that have shown associations between social factors, including isolation (e.g., removing an animal from a group [8]), instability (e.g., changing a group’s social structure [9]), and crowding (e.g., limiting space for access to resources [10]), with compromised immune function and clinical signs of disease. However, for intensively managed dairy cows less research has focused on the social environment before calving and how it may relate to disease risk, despite many of these social changes happening simultaneously (e.g., moving cows between social groups and having to compete for resources).

Research to date on the impact of the social environment on dairy cows has focused on determining group-level changes in behavior in response to changes in management (e.g., [11]). For example, cows forced to compete for access to feed before calving have higher agonistic behavior and a higher rate of feed intake before calving compared to cows that were fed without competition [11]. Moving cows between social groups has also been found to cause a decline in both feeding time [12] and feed intake [13]. These changes in feeding behavior may increase a cow’s risk of disease after calving (e.g., [14]), but little research has investigated this relationship at the cow level. Only a few studies have considered differences between individuals (e.g., [15]) and the consistency of behavioral characteristics (e.g., [16]) in dairy cows.

Little research has investigated the relationship between management, behavior, and disease risk in dairy cattle. Proudfoot et al. [17] found that an unpredictable and competitive social environment before calving increased agonistic behavior at the feed bunk, feeding rate, and the risk of endometritis in the three to five weeks after calving in multiparous dairy cows. Endometritis is characterized by endometrial inflammation present ≥ 21 d after calving and without systemic signs of illness [18]. Although no research to our knowledge has measured the effect of endometritis on cow behavior, there is evidence that cows with metritis, a similar illness, can impact feeding, social, and lying behavior in the days before diagnosis [19].

No studies to our knowledge have investigated the relationship between individual differences in behavior in response to changes in the social environment and health outcomes in cattle. The objectives of this study were to (1) determine if cows show consistent individual differences in dry matter intake (DMI), feeding behavior, and social behavior in the four weeks before calving in different social environments, and (2) determine the relationship between individual differences in behavior and disease (endometritis) in dairy cows housed under these conditions. We predicted that cows would have stable individual differences, and that differences in DMI, feeding behavior, and social behavior in the weeks before calving would be associated with cytological endometritis after calving. This paper is part of a larger study that focused on the effects of social stress during the prepartum period on transition cow behavior and health (see [17] for treatment effects).

## 2. Materials and Methods 

The experiment was conducted between September 2012 and April 2013 at The University of British Columbia’s Dairy Education and Research Centre in Agassiz, British Columbia. Cows were cared for according to the guidelines provided by The Canadian Council on Animal Care (2009) and protocol #A14-0040 approved by The University of British Columbia’s Animal Care Committee according to the guidelines provided by the Canadian Council on Animal Care.

A general description of methods and detailed treatment descriptions can be found in [17]. Briefly, 16 groups of 4 Holstein dairy cows were used in this experiment (n = 64). Each group included 1 primiparous cow and 3 multiparous cows. All experimental cows were previously housed in one of two group free-stall pens in the same building as the experimental pens. Cows were assigned to groups based on their expected calving dates to make the group assignments as random as possible. Groups were moved into 1 of 4 experimental pens approximately 5 weeks before the average expected calving date of the cows in each group. To control for seasonal effects, groups from both treatments were moved into the pen at approximately the same time; when each group was finished with the experiment (i.e., when all of the cows had calved), a new group was added to the pen until all 16 groups completed the experiment.

Experimental pens included 12 lying stalls fitted with mattresses (Pasture Mat, Promat Inc., Woodstock, ON, Canada) covered with 5 cm of sand bedding, vulcanized rubber floors in the alleys and crossovers (Red Bard Dairy Mat, North West Rubber Mats Ltd., Abbotsford, BC, Canada), and 6 gated electronic feed bins (Insentec, Marknesse, Holland). To access the feed bins, cows were required to place their heads over a feed gate so that an antenna could read the unique radio frequency signal in their ear tag (Allflex, High-Performance ISO Half Duplex Electronic ID Tag, Allflex Canada, St-Hyacinthe, QC, Canada). Using a computer connected to the feeding system, experimenters could assign cows to access feed from one or all the feed bins in the pen. Cows were fed a close-up total mixed ration (TMR) ration twice daily at 07:00 and 16:00. TMR samples were collected once weekly and were dried at 60 °C to calculate the DM content of the feed. After calving, cows were moved into one of two free-stall pens.

Each group was housed in an experimental pen starting at 5 weeks before their predicted calving date to collect baseline data. During the baseline phase, all groups had access to 12 lying stalls (3 stalls: 1 cow) and free access to 4 of the 6 feed bins (1 bin: 1 cow).

At 4 weeks before their expected calving dates, 8 of the groups were assigned to a predictable and non-competitive social environment (‘predictable’ treatment). Cows in the predictable groups remained in the same pen and were given free access to all 6 feed bins (1.5 bins: 1 cow) and 12 lying stalls (3 stalls: 1 cow) for the duration of the experiment. These groups were fed at approximately the same time each day (07:00 and 16:00).

The remaining 8 groups were assigned to an unpredictable and competitive social environment (‘unpredictable’ treatment). To create an unpredictable and competitive environment, we used a combination of overstocking at the feeding area, social instability (‘partner change’), and unpredictable feeding times (‘delayed feed access’). In addition, these unpredictable groups were moved into a new pen with 4 non-experimental, multiparous ‘resident’ cows. Each cow could access only one of 4 feed bins (1 bin: 2 cows). Cows had access to 8 lying stalls (1 stall: 1 cow). Resident cows were assigned to access one feed bin each, and this bin assignment remained the same for the duration of the trial. Cows in the unpredictable groups were randomly assigned to access one of the 4 feed bins on the first day they entered the pen, forcing them to compete for feed with one of the resident cows. On the following day, the cows remained assigned to the same feed bin, but at morning feeding each group experienced a random ‘delayed feed access’ of 0, 1, 2, or 3 h. To delay feeding, the bins were filled with fresh TMR at the normal hour (approximately 07:00), but the gates were manually closed until the delayed feed access was complete; all cows in the same group experienced the same delayed feed access. On the third day, cows were reassigned to a new feed bin, which caused a ‘partner change’ and thus they were required to compete with a new resident partner for access to feed. These delayed feed access periods and partner changes were alternated daily until cows showed signs of calving. Two weeks after entering the study, unpredictable groups were subjected to another regrouping event that involved a new pen with 4 new resident cows (‘pen change’). To ensure that no cows habituated to the delayed feed access or bin change regime, the order of bin changes (e.g., which bins cows were assigned to) and the order of delayed feed access times (e.g., whether it was 0, 1, 2, or 3 h) were randomized for the first group using the random function of excel, and this same order was applied to all groups.

Feeding behavior data were automatically collected from the electronic feeding system (validated by [20]). For each visit the electronic feed bins recorded the amount of feed consumed, and the feeding duration during each visit for individual cows. These data were used to calculate the daily DMI (kg/d), daily feeding duration (min/d), average rate of DMI (kg/min), the duration of each visit (min/visit), DMI per visit (kg/visit), as well as the number of visits per day (no./d). The number of visits to the bins per day was further separated into the proportion of visits that were rewarded (i.e., the cow consumed feed during the visit), and unrewarded (i.e., the cow did not consume feed during the visit; ‘non-nutritive’ visit). For a rewarded visit to be detected by the feed bins, the cow must have had access to that feed bin. However, non-nutritive visits were recorded if (1) the cow had access to the feed bin and the gate opened but 0 kg of feed was consumed, or (2) the cow did not have access to the feed bin and she put her head over the gate, but the gate did not open. Cows were also classified has having low or high daily DMI using their average daily DMI over the experimental period and split at the median.

To measure social competition at the feed bins, physical ‘replacements’ at the feed bins were calculated automatically from the feed bin data using methodology described by Huzzey et al. [21]. Briefly, a replacement was considered to occur if a cow was feeding at a bin (‘reactor’), she left the bin, and another cow (‘actor’) used the bin within 26 s of the reactor leaving. These data were summarized into the number of times a cow was involved in a replacement (either as an actor or a reactor) per day.

Blood samples were taken from the coccygeal vein (Vacutainer, Becton Dickinson, Franklin Lakes, NJ, USA) on the last day of the baseline period and once weekly at weeks −2, week −1, and week +1 after calving. Blood sampling occurred at approximately 09:00 on each sampling day. For further methodological details, results, and discussion of blood measures please see [17].

Using the methods described by Dubuc et al. [22], a uterine cytology was performed by a veterinarian 29 ± 6 d after calving for each cow. Cows were considered to have cytological endometritis if ≥ 6% of the cells were identified as neutrophils. For a detailed description of the methods see [17].

Statistical analyses were performed in R (version 3.4.3, R Foundation for Statistical Computing, Vienna, Austria). Of the 64 animals included in this study, 17 were not included in the final analysis (predictable, n = 9; unpredictable, n = 8) because they calved early with twins (n = 2 predictable, n = 2 unpredictable), aborted (n = 1 predictable), were not pregnant (n = 1 unpredictable), calved > 2 weeks early and thus did not allow for the collection of meaningful data (n = 2 predictable, n = 1 unpredictable), or did not have an endometritis exam after calving due to missing equipment (n = 4 predictable, n = 4 unpredictable). The remaining 47 individuals were included in final statistical analyses (predictable, n = 23; unpredictable, n = 24). Daily feeding data automatically collected by the Insentec system were combined into a single data frame that included data for the 4 weeks of the treatment. The data were systematically checked for extreme outliers, which were then investigated using video recordings. Individual outliers were excluded or corrected if video revealed that the data recorded by the Insentec system was incorrect (n = 34 of 124,622 visits). On one day, the air compressor controlling the feed barrier was broken, therefore observations from this day were excluded from the analysis.

To determine if cows showed stable individual differences in feed intake, feeding behavior, and physical replacements during the experimental period, data from week −4, −3, −2, and −1 relative to calving were collapsed by day and by cow, yielding one value per day for each cow. The data from the first 2 weeks (weeks −4 and −3) and second 2 weeks (weeks −2 and −1) of the experimental period were then averaged by cow, yielding one value per cow for each 2 week period. Individual measures of DMI, feeding behavior, and physical replacements were then compared between the first 2 weeks and the second 2 weeks of the experimental period using a Spearman (rank) correlation (ρ). Note that the Spearman correlation was used as it is more robust with non-normally distributed data than the Pearson correlation [23]. Correlation was considered to be significant when *p* < 0.05.

Logistic regression using a logit link was used to test the effect of individual differences in DMI, feeding behavior, and physical replacements on the presence/absence of cytological endometritis (1) across treatments and (2) within each treatment. Daily measures of DMI, feeding behavior, and physical replacements were summarized by cow, yielding one value per cow for each measure. For models predicting the presence/absence of cytological endometritis across treatments the two-way interaction between each measure and treatment were tested and retained in the model if significant (*p* < 0.05).

## 3. Results

Overall, cytological endometritis was detected in 7 of the 23 cows in the predictable treatment and 13 of the 24 cows in the unpredictable treatment. For comparisons between the two treatments see [17].

Cows showed consistent variation in their DMI, feeding behavior, and social behavior between the first two weeks and the second two weeks of the four-week experimental period. Cows were consistent in their total daily DMI (kg/d; ρ = 0.63, *p* < 0.001), average DMI per visit (kg/visit; ρ = 0.91, *p* < 0.001), daily feeding duration (min/d; ρ = 0.56, *p* < 0.001), average DMI rate per visit (kg/min; ρ = 0.66, *p* < 0.001), daily number of visits (no./d; ρ = 0.93, *p* < 0.001), and daily percentage of non-nutritive visits (%; ρ = 0.85, *p* < 0.001). In addition, we found stable individual differences in the number of times cows were involved in social replacements at the feed bins as an actor (ρ = 0.78, *p* < 0.001) and as a reactor (ρ = 0.74, *p* < 0.001).

**Figure 1:**
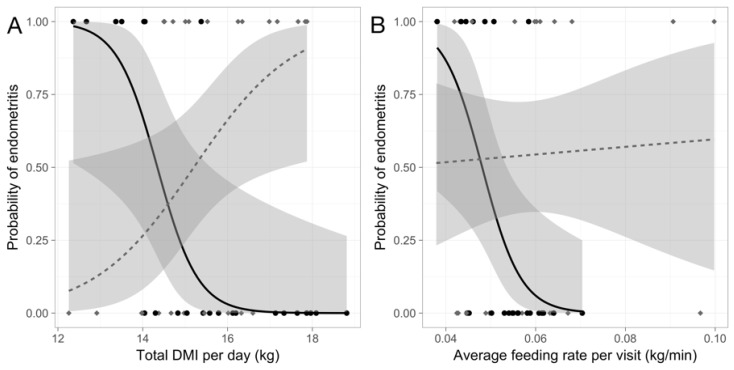
The probability postpartum cytological endometritis given individual differences in (**A**) the total DMI per day (kg) and (**B**) the average rate of DMI per visit (kg/min) during the four weeks before calving in Holstein dairy cows managed in a predictable and non-competitive social environment (black solid line and circles; n = 16 healthy, n = 7 developed endometritis) or an unpredictable and competitive social environment (gray dashed line and diamonds; n = 11 healthy, n = 13 developed endometritis). Gray bands indicate 95% confidence.

Individual differences in DMI affected the risk of developing cytological endometritis differently depending on the social environment (*p* < 0.01; Figure 1; Table 1). Cows in the predictable treatment with consistently low total daily DMI were at greater risk for cytological endometritis (odds ratio (OR) (95% confidence interval): 0.17 (0.02, 0.53); *p* < 0.05). However, for cows in the unpredictable treatment low total daily DMI was protective against cytological endometritis (OR: 1.93 (1.09, 4.14); *p* < 0.05).

The relationship between rate of DMI and disease was also dependent on treatment (*p* < 0.05; Figure 1); a low rate of DMI was a risk factor for disease for cows in predictable groups (OR: 4.2 × 10^−101^ (8.6 × 10^−206^, 4.8 × 10^−30^); *p* < 0.05), but was unrelated to cytological endometritis for cows in unpredictable groups (*p* > 0.8).

The total number of visits to the feed bins per day was not associated with the likelihood of developing cytological endometritis in either treatment (predictable: *p* > 0.5, unpredictable: *p* > 0.7). Across both treatments, the percentage of non-nutritive visits to the feeder was not associated with increased likelihood of cytological endometritis (*p* > 0.1). However, 51% of the cows with a higher than average percentage (>23%) of non-nutritive visits developed cytological endometritis; whereas, only 30% of the cows with a lower percentage of non-nutritive visits developed cytological endometritis. There was also no relationship between the total daily feeding time and the likelihood of cytological endometritis (predictable: *p* > 0.2, unpredictable: *p* > 0.2). The average amount of feed consumed per visit was also unrelated to disease in either of our two treatments (predictable: *p* = 0.09, unpredictable: *p* > 0.5).

Social behavior at the feed bins did not predict disease outcomes in either treatment. Across both treatments, we noted no relationship between disease risk and the number of times a cow replaced others from the feed bins as an actor (*p* > 0.2) or was replaced as a reactor (*p* > 0.4).

## 4. Discussion

To our knowledge, these findings provide the first evidence that cows show consistent individual differences in feeding and social behavior during the four weeks before calving in predictable and non-competitive social environments as well as unpredictable and competitive social environments. We also demonstrate that there is a relationship between individual differences in behavior and disease (cytological endometritis) in dairy cows, but that this relationship is based, in part, on the predictability and competitiveness of the social environment provided.

Given previous research, it was not surprising that low daily DMI before calving increased a cow’s probability of being diagnosed with cytological endometritis in the predictable treatment. Cows in the predictable treatment in the lowest quartile for average daily DMI consumed higher amounts of dry matter (DM) compared to previous reports for cows diagnosed with subclinical metritis and endometritis (tie-stall [24]; free-stall [14]). Differences in housing type and season may have contributed to differences in intake between these studies. That low DMI prepartum affects disease risk postpartum in dairy cows is not new; for example, Hammon et al. [24] provided evidence that cows housed in tie-stalls that develop subclinical endometritis after calving have lower DMI in the peripartum period, leading to decreased immune function after calving. While the majority of research is in agreement that low DMI prepartum is associated with higher risk of disease post-partum, recent evidence has been unable to replicate these prepartum feeding behavior differences between healthy and metritic cows post-partum [25].

It was surprising that the relationship between DMI and cytological endometritis was reversed for cows housed in the unpredictable treatment; cows with high DMI had a higher probability of developing cytological endometritis compared to those with lower DMI. Cows in the unpredictable treatment were subjected to a highly competitive social environment at the feed bunk, a known stressor for dairy cows [26]. Reducing the available feed bunk space per cow in free-stall systems has been shown to increase feed intake in dairy cows [26,27].

Several studies in rodents and humans have found that social stressors can alter feed intake, but the direction of the effect is driven by the type and duration of stressor, as well as the individual’s reactivity to stress (reviewed by Torres and Nowson [28]). In humans, an acute stressor of short duration has been shown to decrease appetite [29]; whereas, a prolonged stressor has been shown to increase appetite, especially for sweet foods [30]. The consumption of sweet foods is considered to be rewarding, so it may be a coping strategy to help mitigate the stressor. For example, Pecoraro et al. [31] found that rats stressed with repeated restraint had decreased activity of the hypothalamic–pituitary–adrenal (HPA) axis when provided with access to palatable food [30]. We predict that the cows housed in our unpredictable treatment with high DMI were more negatively affected by the unpredictable environment, and that the higher feed consumption may have been a behavioral strategy to cope with stress. However, this coping strategy may have led to behavior similar to ‘slug’ feeding, which may have increased the cow’s risk of sub-acute ruminal acidosis (SARA; [32]). The direct link between high intake in an unpredictable environment and disease risk after calving warrants further study.

A low rate of DMI was a risk factor for cytological endometritis for cows in the predictable treatment but was unrelated to cytological endometritis in the unpredictable treatment. In dairy cows, competition at the feed bunk has been shown to affect the rate of feed consumption; cows fed in highly competitive feeding environments have higher rates of DMI compared to those housed in less competitive environments [11]. Subordinate cows increase their feeding rate more than dominant cows in competitive feeding situations [33] but may also avoid competition and aggressive interactions at the feed bunk [34,35]. In the predictable treatment (1.5 bins: 1 cow) cows that ate more slowly may have been subordinate or feeling ill [36,37], both factors potentially contributed to these animals avoiding the feed bunk in the hours after fresh feed delivery when competition for feed was higher despite having access to multiple bins. Waiting until later in the day to eat may have resulted in reduced feed availability and quality due to sorting [38,39]. This strategy may have placed them at greater risk of nutritional deficiencies that disrupt healthy immune function and increase the likelihood of disease. In contrast, waiting until later in the day to eat may not have been a possibility in the unpredictable treatment due to extremely high competition and consumption around fresh feed delivery given that there were two cows assigned to each bin; thus, some cows elected to increase the rate of DMI resulting in high DMI in a shorter period of time. We did not detect a difference in the rate of DMI for the cows in the unpredictable treatment; cows showed greater variation in rate of DMI in the unpredictable compared to cows in the predictable treatment. However, our limited sample size may have prevented the detection of any difference for cows in the unpredictable treatment.

The total number of visits to feed bins and the percentage of those visits that were non-nutritive were unrelated to cytological endometritis. However, despite not being able to differentiate between strategies leading to non-nutritive visits we speculate that there may have been differences in the underlying motivation leading to these types of visits between the two treatments. For cows in the predictable treatment, where competition for feed bins was negligible, this variable was likely driven by feed sampling; cows exploring the variation in feed quality by visiting different bins, accessing the feed bins but electing not to feed from the bin [40]. Unlike cows in the predictable treatment, cows in the unpredictable treatment were only able to eat from one bin in the pen and bin assignment changed every other day, thus three of the four available bins would not open to them. This likely resulted in increased non-nutritive visits as cows sought to locate their bin by putting their heads over the gate. A high frequency of this ‘bin checking’ behavior at the previously assigned bin in the unpredictable treatment could reflect a distinct underlying motivation in the cows. For example, in humans, compulsive checking has been associated with generalized anxiety disorder [41] and in deer mice obsessive-compulsive individuals have altered sociability, being more socially isolative in the presence of non-obsessive-compulsive conspecifics [42]. Alternatively, cows in the unpredictable treatment may have been experiencing frustration as a result of failing to receive food from previously rewarded feed bins [43]. In the unpredictable treatment, the constant changes in feed delivery times as well as which bin they could access to feed likely impacted their ability to predict when and where they could access feed. This loss of perceived control over when and where they would have feed access likely reduced their perceived ability of having some semblance of control over their environment; an aspect that cows are highly motivated to achieve [44] and referred to by others as controlled effectiveness [45]. An unpredictable environment may impair the welfare of the individual animals. Future work should examine this type of checking behavior as a potential indicator of frustration and as a risk factor for disease.

Social replacements were not related to endometritis in our study. One shortcoming of the present study is that the automated algorithm [21] only measured social replacements and thus failed to count instances where a cow was displaced by another cow (actor), but the actor did not enter the bin. Individual differences in social behavior may have been more informative had we recorded more subtle agonistic behaviors at the feed bunk.

## 5. Conclusions

This research provides evidence that there are robust individual differences in intake, feeding, and physical replacements in adult dairy cows. The results of this study also provide the first evidence that individual differences in DMI and rate of DMI in dairy cows are factors to consider when evaluating postpartum disease risk in dairy cows. Furthermore, the social environment acts to moderate the effects of feed intake on the risk for cytological endometritis, though the mechanism remains unclear. Future research should attempt to model these individual differences using personality dimensions that could be linked with the risk for cytological endometritis or other diseases.

## Figures and Tables

**Table 1:** Odds ratio (OR) of postpartum cytological endometritis given the total daily dry matter intake (DMI; kg/d), average DMI per visit (kg/visit), daily feeding duration (min/d), average DMI rate per visit (kg/min), daily number of visits (no./d), percentage of non-nutritive visits (%), and number of social replacements as an actor and reactor per day (no./d) for pregnant Holstein cows in either a predictable and non-competitive social environment (‘predictable’) or an unpredictable and competitive social environment (‘unpredictable’) for the four weeks before calving (predictable: n = 23; unpredictable: n = 24).

Measure	Estimate	Standard Error	Odds Ratio	OR * 95% Confidence Interval		*p*-Value
				Lower	Upper	
Total daily DMI (kg/d) **						
Predictable	−1.77	0.82	0.17	0.02	0.53	0.03
Unpredictable	0.66	0.33	1.93	1.09	4.14	0.04
Average DMI per visit (kg/visit)						
Predictable	−20.52	12.24	<0.01	<0.01	0.35	0.09
Unpredictable	3.59	5.49	36.26	<0.01	6.29 × 10^6^	0.51
Daily feeding duration (min/d)						
Predictable	0.02	0.01	1.02	0.99	1.05	0.26
Unpredictable	0.02	0.01	1.02	0.99	1.04	0.20
Average DMI rate per visit (kg/min) **						
Predictable	−231.13	99.49	<0.01	<0.01	<0.01	0.02
Unpredictable	5.21	24.29	183.09	<0.01	3.76 × 10^24^	0.83
Daily number of visits (no./d)						
Predictable	0.01	0.02	1.01	0.98	1.04	0.57
Unpredictable	−0.01	0.03	0.99	0.93	1.05	0.77
Percentage of non-nutritive visits (%)						
Predictable	0.06	0.07	1.06	0.93	1.23	0.37
Unpredictable	0.167	0.13	1.18	0.93	1.58	0.20
Number of replacements as an actor per day (no./d)						
Predictable	0.05	0.05	1.06	0.95	1.18	0.30
Unpredictable	0.07	0.11	1.07	0.87	1.35	0.54
Number of replacements as a reactor per day (no./d)						
Predictable	0.05	0.05	1.05	0.95	1.17	0.33
Unpredictable	−0.05	0.12	0.95	0.74	1.20	0.65

* OR: Odds Ratio. ** Significant two-way interaction with the predictable and unpredictable treatment on the presence/absence of cytological endometritis (*p* < 0.05).
